# Supplementary material for: CLUES A Comprehensive Workflow for Integrating Geospatial Data in Biomedical Research
Source: Nat Commun. 2026 May 13;17:4330. doi: 10.1038/s41467-026-73048-6 (PMC13172076; doi:10.1038/s41467-026-73048-6)
Supplement: Supplementary file 1 — Supplementary Information [file 41467_2026_73048_MOESM1_ESM.pdf]

Supplementary Information

CLUES A Comprehensive Workflow for Integrating Geospatial Data in Biomedical Research

Marcel Jentsch[1], Elli Polemiti[1,2], Paul Renner[3,4], Sören Hese[3], Kerstin Schepanski[4], Roland Eils[1,2], Andre Marquand[5], Sven Twardziok[1,6], Gunter Schumann[6,7,8,9] on behalf of the environMENTAL consortium

[1] Berlin Institute of Health at Charité - Universitätsmedizin Berlin, Center of Digital Health, 10117 Berlin, Germany.

[2] Health Data Science Unit, Bioquant, Medical Faculty, University of Heidelberg, Im Neuenheimer Feld 267, 69120, Heidelberg, Germany.

[3] Department of Earth Observation, Institute of Geography, Friedrich Schiller University Jena, Leutragraben 1, 07743 Jena, Germany.

[4] Freie Universität Berlin, Department of Earth Sciences, Institute of Meteorology, Carl-Heinrich-Becker Weg 6-10, 12165 Berlin, Germany.

[5] Radboud University Medical Center, Geert Grooteplein Zuid 10, Nijmegen, 6525 GA, Netherlands.

[6] German Centre for Mental Health (DZPG), Berlin-Brandenburg site, 10117 Berlin, Germany

[7] Centre for Population Neuroscience and Stratified Medicine (PONS), Department of Psychiatry and Neuroscience, Charité Universitätsmedizin Berlin, 10117 Berlin, Germany.

[8] Centre for Population Neuroscience and Precision Medicine (PONS), Institute for Science and Technology of Brain-inspired Intelligence (ISTBI), Fudan University, Shanghai 200437, P.R. China

[9] National Center for Neurological Disorders, Huashan Hospital, Fudan University, Shanghai 200437, P.R. China

SI 1. Scalability and Performance evaluation

To assess the computational performance and scalability of the CLUES framework, we conducted a series of tests across varying spatial and temporal extents, and data formats.

First, we assessed CLUES performance on environmental dataset generation using three geographic regions, such as France, Suriname, and Nepal, which differ substantially in size of the corresponding bounding box (France: 1,247,000 km^2, Nepal: 362,000 km^2, Suriname: 210,000 km^2) to demonstrate the applicability of CLUES across diverse geographic scales and global contexts.

For each country, we initially downloaded data for a single year and progressively added additional years. During each step, we recorded the processing time and storage usage.

The initial step required the longest execution time, as it included the download and preparation of both time-invariant datasets (e.g. topography, settlement structure) and time-resolved datasets for the first requested year. Subsequent steps involved only the addition of time-resolved datasets for climate and atmospheric variables.

Panels A and B of Supplementary Figure S1 illustrate the total runtime as a function of the number of processed years for each region and storage requirements respectively.

Two main factors influenced overall performance:

1. Neighbourhood-based spatial computation: In the initial step, neighbourhood metrics are computed over raster grids based on the spatial extent of the requested area. Computational cost increases quadratically with the size of the area, i.e., number of raster cells and kernel size. For example, computing neighbourhood statistics on a 1000×1000-pixel requires processing 1,000,000 pixels. Users can reduce processing time by restricting neighbourhood calculations to locations of interest rather than full spatial coverage.

2. Climate and atmosphere data retrieval**:** Downloads from the Copernicus Data Store are executed sequential due to API constrains, limiting throughput and introducing variability in runtime. As a result, downloading multi-year datasets may take several hours depending on server responsiveness.

Overall, CLUES demonstrates scalable performance for multi-year, multi-country data extraction, with the most computationally intensive operations occurring during the initial setup phase.

In addition, we evaluated the performance of CLUES during the linking step. The two raster formats used within CLUES (GeoTIFF and netCDF) we assessed separately. To systematically assess the performance of spatial linking operations between point locations and raster data, we generated synthetic GeoTIFF datasets with uniformly distributed random pixel values at varying spatial resolutions, all covering an identical geographic bounding box. For each raster dataset, multiple sets of randomly generated point locations with increasing sample sizes were created. Each point lay within the fixed bounding box.

We measured the time required to extract raster values at the point coordinates. The execution runtimes were recorded alongside raster size and sample size. The results are shown in Supplementary Figure S1 panel C. Linking locations to geoTIFFs can be done fast and frugal.

To test the performance of the linking with netCDF files we used a CLUES-generated environmental dataset for the Scandinavian peninsula (bounding box: 1,831,726 km^2) for the period 2000-2025. We generated two sets of point locations, 1000 and 10,000 locations and for each netCDF file, we recorded linking runtime and file size. The results are shown in Supplementary Figure S1 panel D. The linkage running time seems to merely by the ratio of location and file size.

All performance measurements reported here were obtained using a single processing core. In contrast, the production scripts used in the CLUES workflow are fully parallelized, allowing the runtime of the linking procedure to scale inversely with the number of available worker processes. Consequently, when executed in a parallel computing environment, the effective linking time can be reduced proportionally to the degree of parallelism.

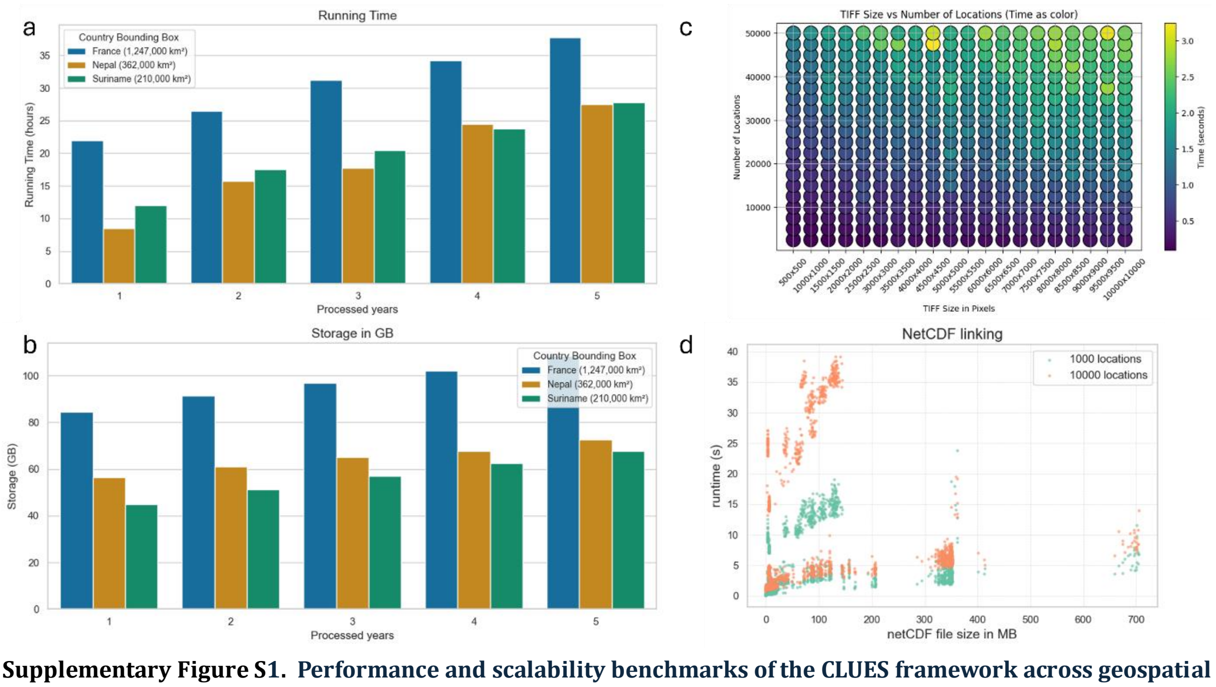

Supplementary Figure S1. Performance and scalability benchmarks of the CLUES framework across geospatial datasets and linking operations. (a) Running time (in hours) for processing 1 to 5 years of data from France, Suriname, and Nepal, illustrating the impact of bounding box size on initial setup time. (b) Storage requirements (in GB) for the same countries and time spans, showing linear growth with additional years and higher demands for larger spatial extents. (c) Linking performance for GeoTIFF files: plot of TIFF size versus number of locations, with colour indicating time per location (s). Results demonstrate efficient spatial lookup even at high resolutions and sample volumes. (d) Linking performance for NetCDF files generated for the Scandinavian peninsula, showing runtime versus file size for two location sets (1000 and 10,000 points). Runtime scales with the ratio between number of locations and the file size. All tests were conducted on a single processing core; production workflows in CLUES are fully parallelized for improved scalability. TIFF; Tagged Image File Format, NetCDF; Network Common Data Form.

SI 2. Configuration management

The CLUES workflow is structured around a set of configuration files that define the database generation. These files include a general workflow configuration file and multiple source-specific configuration files, each serving a distinct function. Together, they specify the core parameters and data products to be downloaded, ensuring the seamless integration and accurate processing of data from various sources, while also support efficient and reproducible data management.

The general workflow configuration file is central in defining the core parameters of the workflow, e.g., designating the storage location of the database. It details the spatial and temporal coverage requirements, thus ensuring that the database is populated with pertinent information. If a CLUES database already exists, the general workflow configuration file also determines which time periods should be updated or added when the database is refreshed.

In addition to the main configuration file, each primary data source is accompanied by its own source-specific configuration file. These files define the datasets to be retrieved from each source, along with source-specific metadata. They include detailed descriptions and parameters, such as URLs and variable names, that guide accurate data download and processing. The source-specific configuration files also specify whether neighbourhood-level processing should be performed for a particular product, and if so, define the type of operation (i.e., mean, std, or Zevenbergen-Thorne) and the corresponding radii of neighbourhood zone to be computed. These processing types are described in detail in the following section. An example of a source-specific configuration file is given below as Copenicus_dem.json.

Each source-specific configuration file is self-contained and specific to its corresponding data source, allowing for easy updates and modifications without disrupting the overall system. This modular design of the configuration system enhances both the flexibility and scalability of CLUES and ensures that new and diverse data sources can be seamlessly integrated. By adjusting the relevant configuration files, users can customize the database to align with their research goals.

Copenicus_dem.json

```
{
    "type":"DEM",
    "format": "geotTiff",
    "variables":[
        {   "name":"Digital_Geospatial_Elevation_Data_30m",
            "url":"https://prism-dem-open.copernicus.eu ...",
            "resolution":"30",
            "neighborhood":{
                "mean":[500,1000],
                "std":[500,1000],
                "zevenbergen_thorne: "yes"}
      ]
}
```
