# Supplementary material for: CLUES A Comprehensive Workflow for Integrating Geospatial Data in Biomedical Research
Source: Nat Commun. 2026 May 13;17:4330. doi: 10.1038/s41467-026-73048-6 (PMC13172076; doi:10.1038/s41467-026-73048-6)
Supplement: Supplementary file 2 — Reporting Summary [file 41467_2026_73048_MOESM2_ESM.pdf]

nature portfolio

Corresponding author(s): Sven O. Twardziok

Last updated by author(s): 21/03/26

Reporting Summary

Nature Portfolio wishes to improve the reproducibility of the work that we publish. This form provides structure for consistency and transparency in reporting. For further information on Nature Portfolio policies, see our Editorial Policies and the Editorial Policy Checklist.

Statistics

For all statistical analyses, confirm that the following items are present in the figure legend, table legend, main text, or Methods section.

n/a	Confirmed	
☒	☐	The exact sample size (*n*) for each experimental group/condition, given as a discrete number and unit of measurement
☒	☐	A statement on whether measurements were taken from distinct samples or whether the same sample was measured repeatedly
☒	☐	The statistical test(s) used AND whether they are one- or two-sided<br>*Only common tests should be described solely by name; describe more complex techniques in the Methods section.*
☒	☐	A description of all covariates tested
☒	☐	A description of any assumptions or corrections, such as tests of normality and adjustment for multiple comparisons
☐	☒	A full description of the statistical parameters including central tendency (e.g. means) or other basic estimates (e.g. regression coefficient) AND variation (e.g. standard deviation) or associated estimates of uncertainty (e.g. confidence intervals)
☒	☐	For null hypothesis testing, the test statistic (e.g. *F*, *t*, *r*) with confidence intervals, effect sizes, degrees of freedom and *P* value noted<br>*Give P values as exact values whenever suitable.*
☒	☐	For Bayesian analysis, information on the choice of priors and Markov chain Monte Carlo settings
☒	☐	For hierarchical and complex designs, identification of the appropriate level for tests and full reporting of outcomes
☒	☐	Estimates of effect sizes (e.g. Cohen's *d*, Pearson's *r*), indicating how they were calculated

Our web collection on statistics for biologists contains articles on many of the points above.

Software and code

Policy information about availability of computer code

Data collection	https://github.com/BIH-DMBS/CLUES, python [rasterio, pyproj, numpy, pandas, geopandas, shapely, cdsapi, xarray, rioxarray, netCDF4, owslib ,ipykernel, ipywidgets, matplotlib, filelock, seaborn, scikit-image, beautifulsoup4, h5netcdf, rasterstats, glob2, bs4, snakemake, pyarrow, fastparquet, cloudscraper, mkdocs, mkdocs-material, mkdocs-macros-plugin, dask, worldpoppy, earthaccess, cfgrib, pyyaml, tomli, pulp==2.7.0, pyhdf], Docker
Data analysis	n/a

For manuscripts utilizing custom algorithms or software that are central to the research but not yet described in published literature, software must be made available to editors and reviewers. We strongly encourage code deposition in a community repository (e.g. GitHub). See the Nature Portfolio guidelines for submitting code & software for further information.

Data

Policy information about availability of data

All manuscripts must include a data availability statement. This statement should provide the following information, where applicable:

- Accession codes, unique identifiers, or web links for publicly available datasets
- A description of any restrictions on data availability
- For clinical datasets or third party data, please ensure that the statement adheres to our policy

No new datasets were generated during this study. CLUES retrieves publicly available environmental datasets from third-party providers, and all data sources used in this workflow are openly accessible. Where available, DOIs are provided for each dataset in the CLUES documentation, along with access links and licensing

information for each data source.

Research involving human participants, their data, or biological material

Policy information about studies with human participants or human data. See also policy information about sex, gender (identity/presentation), and sexual orientation and race, ethnicity and racism.

Reporting on sex and gender	n/a
Reporting on race, ethnicity, or other socially relevant groupings	n/a
Population characteristics	n/a
Recruitment	n/a
Ethics oversight	n/a

Note that full information on the approval of the study protocol must also be provided in the manuscript.

Field-specific reporting

Please select the one below that is the best fit for your research. If you are not sure, read the appropriate sections before making your selection.

☒ Life sciences ☐ Behavioural & social sciences ☐ Ecological, evolutionary & environmental sciences

For a reference copy of the document with all sections, see nature.com/documents/nr-reporting-summary-flat.pdf

Life sciences study design

All studies must disclose on these points even when the disclosure is negative.

Sample size	n/a (There was no predetermined number of datasets. Instead, selection was guided by the following criteria: (i) global coverage to ensure harmonisation across regions; (ii) optimal spatial and temporal resolution; and (iii) comprehensive representation of key environmental domains including urban and natural space, climate, air pollution, and regional socioeconomic status (SES). For each variable, the dataset with the best available resolution and coverage was selected. While additional global SES datasets exist (e.g. World Bank, UNdata), their limited spatial and temporal resolution precluded their inclusion at this stage. As a result, SES variables are currently limited to European countries, for which harmonised regional data are available. The selected datasets are sufficient for the intended purpose of CLUES, which is to provide researchers with a standardised, GDPR-compliant workflow for linking environmental exposures to participant locations across a wide range of epidemiological and biomedical studies. As new environmental datasets become available with improved global coverage and resolution, CLUES will be continuously expanded to incorporate them.)
Data exclusions	n/a (No data were excluded from the analyses. CLUES is a workflow for downloading and linking publicly available environmental datasets; all data retrieved from the integrated third-party sources are included in the database without exclusion. The only exception is when specific datasets are not available for a requested region or time period due to the limitations of the primary data source itself, in which case no data is downloaded for that variable. These limitations are inherent to the primary data sources and not the result of exclusion criteria applied by CLUES.)
Replication	n/a (CLUES is a computational workflow and does not involve experimental findings. Reproducibility is ensured by design through the following: (i) all code is publicly available on GitHub (https://github.com/BIH-DMBS/CLUES/) with a permanent Zenodo DOI (10.5281/zenodo.19092838); (ii) the workflow is fully automated using Snakemake, ensuring that all steps are executed in a standardised and reproducible manner; (iii) all third-party data sources are publicly accessible, allowing any researcher to reproduce the database generation independently; (iv) detailed documentation and Jupyter notebooks are provided to guide users through all steps of the workflow. Any variability in results is expected to be limited to differences in data availability from third-party sources at the time of download, which is inherent to the nature of continuously updated environmental datasets.)
Randomization	n/a (Sample allocation into experimental groups was not relevant to this study. CLUES is a computational workflow that does not involve experimental groups, sample allocation, or covariates. The performance and scalability benchmarks presented in the supplementary information were conducted using predefined geospatial datasets and linking operations, with no allocation or randomisation required.)
Blinding	n/a (Blinding was not relevant to this study. CLUES is a computational workflow for downloading and linking publicly available environmental datasets and does not involve any experimental groups, treatment allocations, or outcome assessments that would require blinding.)

Reporting for specific materials, systems and methods

We require information from authors about some types of materials, experimental systems and methods used in many studies. Here, indicate whether each material, system or method listed is relevant to your study. If you are not sure if a list item applies to your research, read the appropriate section before selecting a response.

Materials & experimental systems

n/a	Involved in the study
x	Antibodies
x	Eukaryotic cell lines
x	Palaeontology and archaeology
x	Animals and other organisms
x	Clinical data
x	Dual use research of concern
x	Plants

Methods

n/a	Involved in the study
x	ChIP-seq
x	Flow cytometry
x	MRI-based neuroimaging

Plants

Seed stocks	n/a
Novel plant genotypes	n/a
Authentication	n/a
